# GSNOR regulates cardiomyocyte differentiation and maturation through protein S-nitrosylation

**DOI:** 10.20517/jca.2021.25

**Published:** 2021-10-13

**Authors:** Zachary W. Grimmett, Nicholas M. Venetos, Richard T. Premont, Jonathan S. Stamler

**Affiliations:** 1 Department of Pathology, Case Western Reserve University School of Medicine, Cleveland, OH 44106, USA.; 2 Institute for Transformative Molecular Medicine, Department of Medicine, Case Western Reserve University School of Medicine, Cleveland, OH 44106, USA.; 3 Department of Biochemistry, Case Western Reserve University School of Medicine, Cleveland, OH 44106, USA.; 4 Harrington Discovery Institute, University Hospitals Cleveland Medical Center, Cleveland, OH 44106, USA.

## Abstract

S-nitrosoglutathione reductase (GSNOR) is a denitrosylase enzyme responsible for reverting protein S-nitrosylation (SNO). In this issue, Salerno *et al.*^[1]^ provide evidence that GSNOR deficiency - and thus elevated protein S-nitrosylation - accelerates cardiomyocyte differentiation and maturation of induced pluripotent stem cells (iPSCs). GSNOR inhibition (GSNOR^−/−^ iPSCs) expedites the epithelial-mesenchymal transition (EMT) and promotes cardiomyocyte progenitor cell proliferation, differentiation, and migration. These findings are consistent with emerging roles for protein S-nitrosylation in developmental biology (including cardiomyocyte development), aging/longevity, and cancer.

## GSNOR-DEPENDENT S-NITROSYLATION REGULATES GSK-3β FUNCTIONS

GSNOR modulates dynamic denitrosylation of hundreds of protein substrates (among > 20,000 in the published literature^[2]^) in response to a variety of biological stimuli^[3]^; Salerno *et al.*^[1]^ add GSK-3β (glycogen synthase kinase-3β) to this list of SNO substrates. GSK-3β is an essential kinase with multiple roles, particularly in cardiovascular physiology^[4]^. Recently, GSK-3β has been shown to be S-nitrosylated at three major sites (Cys76, Cys199, and Cys317) and multiple minor sites, leading to inhibition of cytosolic kinase activity (with subsequent translocation to the nucleus and phosphorylation of nuclear targets - in lieu of cytoplasmic targets)^[5]^. S-nitrosylation of GSK-3β inhibits kinase activity independent of canonical inhibitory Ser9 phosphorylation, providing a novel locus of regulation for this important kinase. Salerno *et al.*^[1]^ innovatively demonstrate that S-nitrosylation of GSK-3β is enzymatically regulated by GSNOR in iPSCs during early cardiomyocyte differentiation. Further, they show that GSNOR^−/−^ iPSCs - with elevated S-nitrosylation - undergo accelerated cardiomyogenesis, including expedited differentiation, proliferation, migration, and EMT compared to wild-type iPSCs. SNO-GSK-3β may influence many of these processes, particularly EMT, which is promoted by three transcription factors (*Snail*, *Slug,* and *Twist*) already known to be regulated by GSK-3β^[6]^. The authors correlate elevated SNO-GSK-3β levels with accelerated cardiomyogenesis in GSNOR^−/−^ iPSCs. Limitations of the study include that the authors did not identify the GSNOR-regulated SNO site(s) on GSK-3β in their model, nor did they perform studies demonstrating a causal role for SNO-GSK-3β in promoting cardiomyocyte differentiation (e.g., wild-type *vs.* SNO-site mutant GSK-3β restoration in knockout iPSCs). Indeed, no published study has yet investigated functional consequences of individual GSK-3β Cys residue S-nitrosylation, which are likely tightly regulated by specific nitrosylase and denitrosylase enzymes *in vivo*^[7]^. Thus, *in vitro* treatment with exogenous NO donors (GSNO^[1]^, CysNO^[5]^, *etc.*) may not always recapitulate physiology. Therefore, further confirmatory studies identifying the major site(s) of SNO regulation of GSK-3β relevant to cardiomyocyte maturation, including utilizing single Cys-mutant GSK-3β constructs combined with functional analyses, would be an illuminating area of future research.

GSNOR-dependent SNO-targets beyond GSK-3β may contribute to altered cardiomyocyte differentiation, and denitrosylases other than GSNOR may play important roles. GSNOR is one of seven known denitrosylases whose purview and targets are largely unexplored. Furthermore, many additional SNO-regulatory enzymes remain to be discovered, including S-nitrosylases (SNO synthases and transnitrosylases) and denitrosylases, including but not limited to GSNOR subtypes; in other words, the multiplex enzyme systems^[7]^ regulating protein S-nitrosylation in cardiomyocytes have yet to be fully uncovered. This complexity elevates the impact of the findings presented by Salerno *et al.*^[1]^, because it links cardiomyocyte differentiation specifically to GSNOR, one of multiple denitrosylase enzymes. In future studies it will be intriguing to explore what role other SNO-processing enzymes may plausibly play in cardiac regeneration and within the purview of stem cell maintenance and proliferation more broadly. Most notably, the SCoR/AKR1a1 denitrosylase has been implicated in metabolic reprogramming that may promote cancer and cellular proliferation^[8]^, and nitrosylase enzymes such as Hcp analogues^[7]^ may have important regulatory functions in these systems, which are entirely unexplored.

## S-NITROSYLATION IMPACTS MULTIPLE PATHWAYS TO REGULATE CARDIOVASCULAR PHYSIOLOGY

Nitric oxide has long been deemed cardioprotective, particularly in the context of ischemia/reperfusion injury^[9]^. Many proteins (including RyR2, HIF-1α, SERCA2, hemoglobin, matrix metalloproteinase 9, complex 1, *etc.*^[3]^) have been shown to be S-nitrosylated in the heart under various conditions - regulating diverse signaling pathways including hypoxic responses, apoptosis, calcium handling/arrhythmogenesis, and microvascular control, among many others^[1]^. Functionally, protein S-nitrosylation exerts cardioprotective effects through diverse mechanisms, including induction of ischemic preconditioning and antioxidative defenses^[9]^. Though S-nitrosylation was originally thought to be broadly cardioprotective^[9]^, recent advances have exposed roles in cardiac pathology. For example, previous work by this laboratory has linked GSNOR to altered adrenergic responses in the heart and periphery^[10]^. GSNOR^−/−^ mice, in addition to exhibiting reduced peripheral vascular tone, demonstrate impaired β-agonist-induced inotropy; because a hallmark of heart failure (with reduced ejection fraction) is loss of inotropic reserve, elevated SNO-protein level is evidently pathologic in this context. Accumulating evidence also points to a causal role for SNO-proteins in heart failure with preserved ejection fraction. By contrast, findings presented by Salerno *et al.*^[1]^ ascribe a beneficial role to elevated SNO-proteins in the heart, at least during early differentiation and maturation. This apparent contradiction reveals a more nuanced role for S-nitrosylation in cardiac physiology - one that is likely dependent on differentiation state, cell type, pathology, presence or absence of β-adrenergic stimulation, *etc.*, and in which both over- and under-production of SNO-proteins is pathologic. In this issue, Salerno *et al.*^[1]^ describe a beneficial role for GSNOR^−/−^ in cardiac development, plausibly dependent upon elevated SNO-GSK-3β. Inhibition of GSK-3β promotes DNA synthesis, cell cycle re-entry and proliferation^[11]^, which may well explain the accelerated iPSC-derived cardiomyocyte development that they observe^[1]^. Unraveling this intricate relationship between S-nitrosylation and cardiovascular physiology will require attention to distinct SNO targets in different pathophysiological contexts.

## GSNOR AND S-NITROSYLATION: IMPACT ON DIFFERENTIATION AND IMPLICATIONS FOR AGING/LONGEVITY

This study also sheds light on the influence of protein S-nitrosylation in stem cell biology. Maintenance of stem cell pluripotency, as well as the process of differentiation, are both regulated by NO; low NO levels suppress differentiation - marked by *Nanog*, *Oct4*, and *Sox2* levels^[12]^ - while high concentrations of NO induce differentiation^[13]^. “Low” and “high” NO likely reflect different SNO targets. Indeed, Salerno *et al.*^[1]^ observe that GSNOR^−/−^ downregulates both *Oct4* and *Sox2*, consistent with accelerated differentiation via “high NO”. In corroborating work^[14]^, epigenetic downregulation of GSNOR appears to drive aging and cell senescence, with a reduction in GSNOR expression evident both in primary cells undergoing senescence as well as in aging mice and humans throughout their respective lifespans. Intriguingly, GSNOR^−/−^ mice also demonstrate features overlapping those of experimental aging models, including impaired DNA repair, deficient osteogenesis, and neuromuscular dysfunction^[14]^. Indeed, GSNOR^−/−^ mice show nitrosative stress in conjunction with mitochondrial dysfunction, due at least in part to S-nitrosylation of specific target proteins (i.e., Drp1 and Parkin) that regulate mitochondrial dynamics and mitophagy^[14]^. Together, these data suggest that the role and function of GSNOR may vary with the age of the organism, in different organs, and across health and disease.

In a recent and exciting advance by Yi *et al.*^[15]^, GSNOR deficiency has also been shown to lead to abnormal hematopoietic stem cell (HSC) regeneration. In response to chemotherapy-induced injury, GSNOR^−/−^ HSCs demonstrate impaired self-renewal and thus diminished reconstitutive capacity relative to wild-type HSCs; this deficit is prevented by inhibition of NO synthesis. Thus, GSNOR-dependent regulation of SNO-protein levels appears to promote maintenance of not only iPSC pluripotency^[1]^ but also HSC self-renewal (and thus the ability to reconstitute the entire hematopoietic system). Unfortunately, Yi *et al.*^[15]^ do not identify SNO-protein substrates of GSNOR that regulate HSC self-renewal - a compelling research direction for future studies investigating aging and longevity.

**Figure 1.**
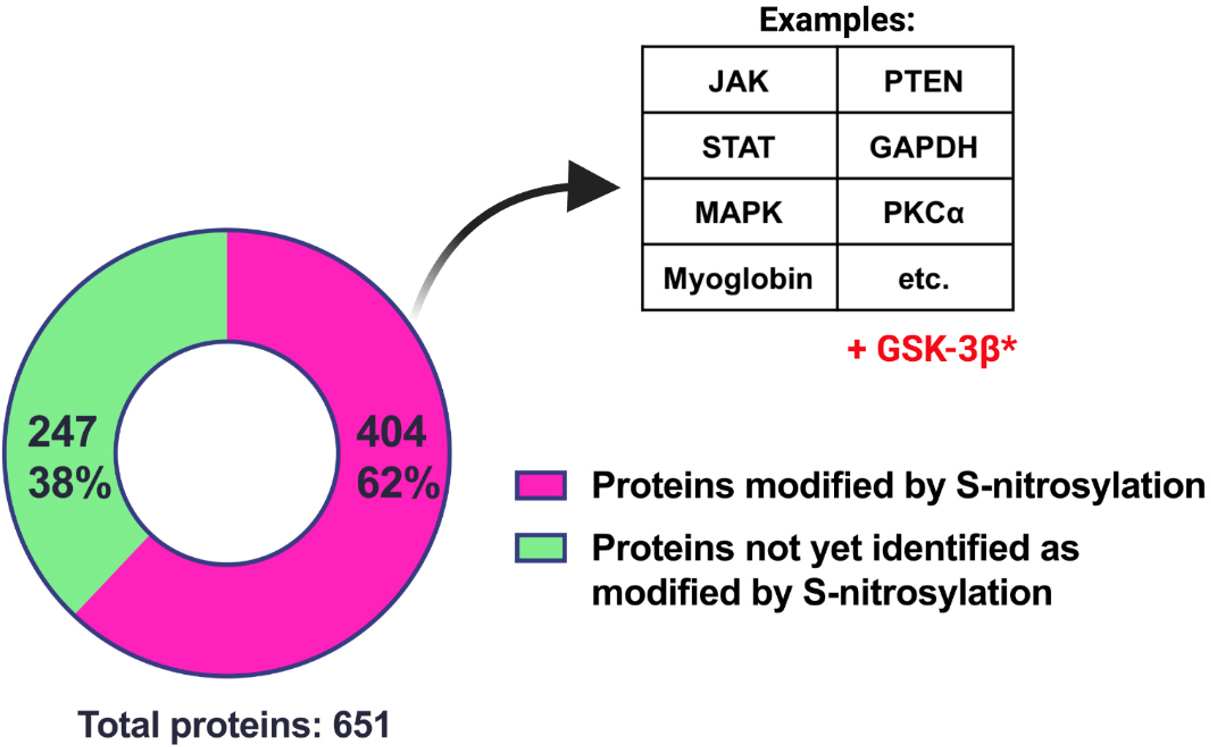
S-nitrosylation modifies a majority of proteins related to aging and longevity. Of 651 plasma proteins identified as significantly associated with age (either over-or under-represented in aged individuals)^[16]^, 404 are known to be S-nitrosylated. Notable examples are displayed in the table to the right. A new addition, GSK-3β, has been identified by Salerno *et al.*^[1]^ in this issue.

Utilizing a dataset containing approximately 25,000 SNO sites identified in > 10,000 proteins compiled by our laboratory, we find that many proteins previously identified as relevant to aging/longevity signaling pathways^[16]^ are S-nitrosylated [Figure 1], though functional outcomes of this S-nitrosylation remain uncharacterized for most targets. Excitingly, Figure 1 contains only proteins identified in the plasma of test subjects; there are therefore many additional S-nitrosylated targets relevant to the aging process that are not among these 651 plasma proteins, including GSK-3β. In cardiac cells specifically, hundreds of proteins are involved in aging/longevity and pluripotency/differentiation, and further characterization of SNO-GSK-3β may expose distinct consequences of this modification in the context of aging and longevity. For example, GSK-3β is known to negatively regulate the transcriptional coactivator PGC-1α, which influences many aspects of energy metabolism^[17]^. One such target of PGC-1α is mitochondrial biogenesis - dysfunction of which has been implicated in aging. Indeed, upregulation of PGC-1α in *Drosophila* is associated with enhanced mitochondrial biogenesis and increased lifespan^[17]^. Thus, SNO-mediated inhibition of GSK-3β might provide an avenue to upregulate PGC-1α and enhance lifespan, which may be counterbalanced by excessive S-nitrosylation of mitochondrial proteins wherein GSNOR deficiency accelerates cellular aging and senescence^[14,17]^.

It thus appears likely that proper *regulation* of protein S-nitrosylation - as opposed to the absolute quantity of NO/SNO - is deficient in aging, and thus, conversely, may drive longevity. Studies aiming to develop therapies focused on maintaining proper regulation of protein S-nitrosylation, particularly GSNOR-regulated S-nitrosylation, throughout the aging process may be illuminating and impactful in enhancing the longevity of organ systems. Additionally, unbiased mass-spectrometry-based studies identifying S-nitrosylated cardiac proteins that regulate differentiation and maturation will be informative for future regenerative therapies, as no such dataset currently exists. Ideally, this valuable data will prove instructive for improving clinical modulation of cardiac regeneration post-injury, and will advance our scientific understanding of cardiac differentiation with the ultimate goal of improving cardiac tissue viability.
